# Tocotrienol Nanoemulsion Platform of Curcumin Elicit Elevated Apoptosis and Augmentation of Anticancer Efficacy against Breast and Ovarian Carcinomas

**DOI:** 10.3390/ijms17111792

**Published:** 2016-10-26

**Authors:** Nelson Steuber, Kathy Vo, Ritambhara Wadhwa, Jordan Birch, Paulina Iacoban, Pedro Chavez, Tamer A. Elbayoumi

**Affiliations:** 1 Department of Pharmaceutical Sciences/Nanomedicine Center of Excellence in Translational Cancer Research (Nanomedicine COE-TCR), College of Pharmacy-Glendale, Midwestern University, Glendale Hall 236-14, 19555 N. 59th Ave., Glendale, AZ 85308, USA; njsteuber@gmail.com (N.S.); piacob@midwestern.edu (P.I.); 2 Arizona College of Osteopathic Medicine, Midwestern University, 19555 N. 59th Ave., Glendale, AZ 85308, USA; kvo95@midwestern.edu (K.V.); rwadhwa96@midwestern.edu (R.W.); jordan.birch1@gmail.com (J.B.); 3 Department of Biomedical Sciences, College of Health Sciences, Midwestern University, 19555 N. 59th Ave., Glendale, AZ 85308, USA; pchave@midwestern.edu

**Keywords:** tocotrienols, tocopherols, polyphenols, nanoemulsion, antiproliferative, biocompatibility, apoptosis, tumor necrosis factor-α, caspase

## Abstract

Vitamin E (VE) tocotrienols (T3), recognized for their cancer-specific anti-proliferative and pro-apoptotic activities, have been previously fabricated into bio-active nanoemulsion (NE) formulations. Here, our viscosity-adapted δ-T3 NE platform was developed to additionally incorporate curcumin (CUR), which is known for its potent suppression of signaling pathways involved in malignant cell growth, survival and metastasis. Thanks to efficient 70:30 wt % surfactant mix of Lutrol F-127:VE-TPGS, in conjunction with optimal CUR loading, a prototype CUR in δ-T3 NE was successfully prepared. Model CUR/δ-T3 NE demonstrated excellent nano-scale aspects (mean particle size = 261 nm, PDI = 0.27, and ζ-potential = −35 mV), pharmaceutical stability, and controlled release properties. Suitability for systemic administration was also verified via standardized in vitro biocompatibility and hemocompatibility assays. In two human cancer cells (MCF-7 and OVCAR-8), our CUR/δ-T3 NE prominently suppressed constitutive NF-κB activation, and significantly induced apoptosis. Finally, the combined CUR/δ-T3 NE produced superior cytotoxicity profiles, in concentration- and time-dependent manners (*p* ≤ 0.05), at least three to four folds lower IC_50_ than in closest CUR control. The strong synergism, estimated in both cultured carcinomas, revealed the augmented therapeutic efficacy of our CUR/δ-T3 NE combined platform, supporting its strong potential towards pharmaceutical development for cancer therapy.

## 1. Introduction

Curcumin (CUR, 1,7-bis[4-hydroxy-3-methoxyphenyl]-1,6-heptadiene-3,5-dione) is a naturally-occurring polyphenol extracted from the rhizome of turmeric, *Curcuma longa* [1,2]. CUR has a long history of use as an Asian spice and in traditional medicines, and has gained vast popularity, within the past few decades, as a potential “natural multi-purpose drug” [3]. For years, CUR was considered a powerful anti-inflammatory agent for the prevention and treatment of many chronic pathological conditions such as; neurodegenerative, cardiovascular, pulmonary, metabolic, autoimmune and neoplastic diseases [1,4]. Mover, CUR has been found to promote wound healing, and reduce cataract formation, as well as protect against pulmonary toxicity and hepatocellular injury. Recently, an expanding body of preclinical and clinical evidence has indicated the potent anticancer activity of CUR, supporting its potential application in in chemoprevention and treatment of a wide variety of tumors including head and neck, lung, colorectal, breast, gastrointestinal, genitourinary, melanoma, neurological and sarcoma [3]. Extensive studies have shown that CUR has a pleiotropic therapeutic effect in cancer, and exerts its anti-cancer effect by various mechanisms [2,4]. The anticancer properties of CUR have been primarily attributed to its restrictive action against tumor necrosis factor-α (TNF-α) canonical pathway, at both transcriptional and post-transcriptional levels, mediated via CUR directly blocking nuclear factor κB (NF-κB), which is a master regulator of inflammation, cell proliferation, apoptosis and angiogenesis [2,5]. Mechanistically, CUR induces apoptosis in neoplastic cells by inhibiting numerous signaling pathways involved in tumor cell proliferation, such as AP-1, and STAT pathways, as well as a group of key kinases including protein kinase C, serine/threonine kinase (AKT), epidermal growth factor receptor (EGFR), extracellular signal-regulated kinase (ERK), and mitogen-activated protein (MAP) kinase in various cancer types [2]. The immense therapeutic value of such broad anti-neoplastic effect of CUR can only be realized when administered via an effective pharmaceutical delivery system [3,4].

While CUR has an excellent safety profile, it has shown limited therapeutic efficacy in past clinical trials, largely due to its low solubility, combined with extensive chemical instability in aqueous buffers, which lead to poor oral absorption, rapid intestinal and hepatic metabolism and rapid elimination, generally manifested as very short plasma half-life (*T*_1/2_ < 0.5 h) [3,5]. Such major challenges to CUR delivery are primarily attributable to its hydrophobic nature and thus extremely low water solubility of the free drug (0.4 ng/mL in physiological pH 7.4) [4,6]. As a class II drug in the Biopharmaceutical Classification System (BCS), CUR suffers from very limited systemic bioavailability, while exhibiting appreciable permeability across biological membranes, thanks to its significant lipophilicity (log *p* = 2.5) [3,6]. Therefore, it has been realized that regardless of the intended route of administration, the free form of CUR suffers from extremely poor systemic bioavailability, diminished plasma levels, and negligible tissue biodistribution, hence insufficient and ineffective cytotoxic concentration in the tumor [3,4,5].

Both Vitamin E isoforms, tocopherols (Tph) and Tocotrienols (T3), contain four derivatives: α, β, γ, and δ, which differ from each other based on the number and position of methyl groups attached to their chromane rings. Recent studies suggest that T3s, which are the unsaturated forms of Vitamin E, are a promising class of anti-cancer compounds that inhibit the growth and survival of many cancer cells—including breast, ovarian, prostate, pancreas, liver, and different skin cancers—without impacting normal cells [7,8,9]. Vitamin E (VE), including T3 analogues, has been shown to be a highly useful compound to encapsulate and enhance the delivery of poorly water-soluble drugs, such as genistein (Gen), quercetin, and other active polyphenols in green tea [10,11]. Effectively, T3-analogues (in particular γ- and δ-T3) have also shown strong potential for cancer chemoprevention and therapy, owing to their potent anti-proliferative and direct mitochondrial pro-apoptotic effects (i.e., specific inhibition of NF-κB, and BCl-2, plus stimulation of JNK/BAX pathways, resulting in mitochondrial uncoupling and cytosolic release of cytochrome *c*) in different solid and hematological tumors [8,12,13,14]. While the notion about the much higher accumulation of T3s in cells, in comparison to Tphs, might contribute to their prominent effects, the diverse mechanisms implicated in T3s action on cancer cell death and other inhibitory pathways are still not fully elucidated [13]. Marked inhibition of pro-survival signaling mediated by the transcription factor NF-κB, in conjunction with activation of the intrinsic pathway of apoptosis, seem to play key roles underlying the anti-neoplastic activity of T3s [7,8]. Therefore, combination of bioactive natural compounds, such as CUR and δ-T3, that targets dysregulated NF-κB signaling along with apoptosis induction would be an important strategy to enhance the cytotoxic efficacy of cancer therapy [9,15,16]. However, the poor solubility and bioavailability profiles of these compounds remain the major challenges to realizing their translational application in the clinic [6,17].

In this regard, many attempts have been conceived for CUR nano-systems to improve the drug’s solubility, enhance its in vivo bioavailability and maximize its biological activity, including, liposomes, solid lipid nanoparticles, niosomes, polymeric nanoparticles, polymeric micelles, cyclodextrins, dendrimers, and silver and gold nanoparticles [3,4,17]. Out of these, a nano-emulsification technique has been quite successful, taking advantage of the lipophilic character of CUR, rather than chemical modification or conjugation [6,18]. Owing to their superior pharmaceutical properties (such as good biocompatibility, biodegradability, physical stability, and ease of large-scale production), optimized nanoemulsion (NE) formulations can incorporate hydrophobic CUR as a payload within its VE-based core matrix [10]. In fact, nano-emulsification of VE compounds has been reported with great success in multiple reports, via the utilization of non-ionic surfactant blends [12,18,19]. Stable NEs enriched with VE compounds (such as γ- and δ-T3s in particular) have been developed for parenteral and oral administration, as nano-delivery vehicles to enhance the systemic bioavailability of many slightly water-soluble chemotherapeutic agents, such as paclitaxel, simvastatin, and Gen [10,20,21,22,23]. VE-based nano-formulations are particularly attractive for the delivery of chemotherapeutics, attributable to the ability of γ-T3 and δ-T3 compounds to sensitize cancer cells towards co-administered therapeutic drugs (e.g., CUR, here) consequently enhancing the overall anticancer efficacy of such combinations.

The aim of our current study was to successfully develop δ-T3 VE-based NE as a pharmaceutical platform not only to improve CUR solubility and enhance its delivery, but to also produce significant augmentation of pharmacological anti-cancer efficacy, evaluated against different cell culture models of human breast and ovarian carcinomas.

## 2. Results and Discussion

Nano-emulsification of active VE compounds has been reported with great success in different reports, mostly via the utilization of non-ionic surfactant blends [14,24]. Stable NEs enriched with Vitamin E (such as α-, γ- and δ-Tph and T3s) have been successfully developed for parenteral and oral administration, initially as antioxidant therapy [24], as well as a nano-delivery vehicle to enhance the systemic bioavailability of numerous slightly water-soluble anticancer agents, such as paclitaxel [22], etoposide, along with natural polyphenols comparable to CUR, such as quercetin, and Gen [18,24,25].

The present communication builds upon our previous reports of hybrid homogenization protocols [12], for optimal hybrid emulsification of T3-based VE oil cores, to yield model NE pharmaceutical platforms [12,26]. We have employed various emulsifying mixtures based on Vitamin E-TPGS (VE-TPGS), or Poloxamer/Lutrol F68 (LF68), as main co-surfactants [19,20], yielding different prototype NE formulations, demonstrating extended shelf-life stability, controlled release profiles of bioactives, and exhibited superior biological activities, in biological test systems [10,12,27].

**Figure 1:**
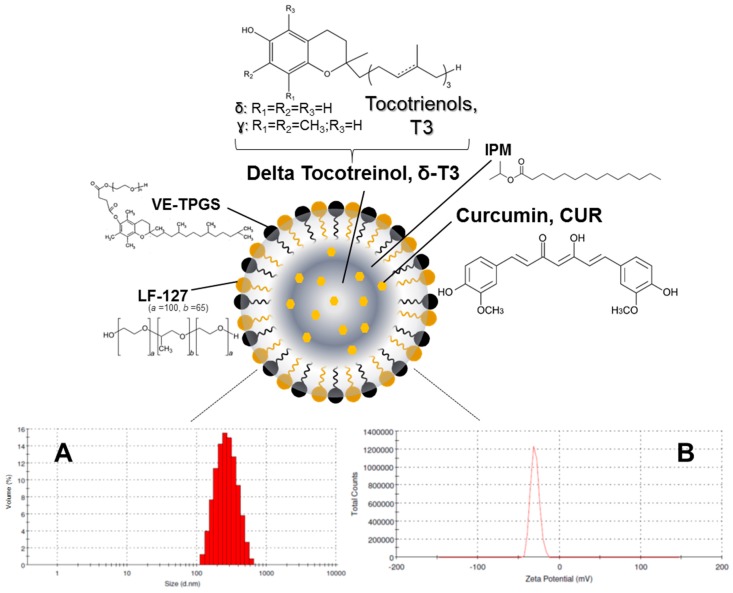
Schematic diagram of tocotrienol nanoemulsion platform of curcumin illustrating the chemical structures of both components of surfactant mixture, Lutrol^®^ F127and Vitamin E TPGS, along with those of the core oil phase, curcumin, and isopropyl myristate. Plus, representative analysis of prototype δ-tocotreinol nanoemulsion loaded with 1.96 ± 0.37 mg/mL of curcumin (*n* = 3), showing mean (**A**) droplet size; and (**B**) interfacial electrical charge (measured as ζ-potential).

Such an optimized system was employed as a model for the development and evaluation of the primary physico-chemical characteristics and pharmaceutical properties of a suitable T3-NE system for enhanced CUR solubilization and stability (Figure 1). Following in vitro biocompatibility and hemocompatibility screens of prototype CUR-loaded δ-T3 NE, its pro-apoptotic and anti-proliferative activities were assessed against different test carcinoma types (here, estrogen-dependent breast and ovarian cancers, MCF-7 and OVCAR-8, respectively, represent two human tumor models in which CUR was repeatedly studied). Gathered data, demonstrating the therapeutic potential of our combined CUR in δ-T3 NE formulation to support anticancer treatment, are discussed in the following sections.

### 2.1. Preparation and Characterization of CUR-Loaded T3-NE Formulation

**Figure 2:**
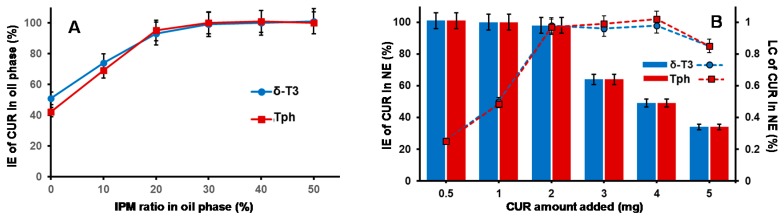
Curcumin incorporation capacity and loading capacity profiles demonstrated for (**A**) δ-tocotrienol oil phase blended with isopropyl myristate; and (**B**) nanoemulsion formulations in respect to amounts of feed curcumin added during preparation (*n* = 3, mean ± SD).

Our currently adopted NE platform composition criteria are derived from our earlier physical and chemical stability data of hybrid T3-NEs, in respect with surfactant blend ratios involving TPGS and/or poloxamers [19,26]. Analogously, the optimal design of a typically viscous T3 oil phase within a Vitamin E-enriched NE platform entailed viscosity modifiers—like isopropryl myristat (IPM) [10,23]—for such oil core composition ratios of T3 ≥ 50 wt %. Blended IPM:T3 oil core wt. ratios (ranging between 50:50-and-20:80) were reported to facilitate the nano-emulsification process (Figure 1), plus successfully supporting the incorporation of coactive therapeutics such as simvastatin [23], and Gen [10]. Indeed, in our current work, Figure 2A reveals gradual improvement in CUR incorporation (IE%) proportional to increasing IPM ratio in the VE (either Tph or δ-T3) oil phase, until it reached almost maximal value at IPM/T3 ratios ≥30 wt %, which also corresponds to reduced dynamic viscosity values of 15–18 mPa·s (vs. ~2400 and ~1700 mPa·s for pure Tph or δ-T3, respectively, data not shown). Hence, a blended IPM:T3 oil phase composition of 30:70 wt % was selected for our NE platform of CUR, as it combines the advantages of low viscosity, feasible hybrid nano-emulsification, and least dilution of the bioactive T3 oil matrix of fabricated NE [10,27].

Effective hybrid nano-emulsifiation of 30 wt % IPM blended VE oil phase (either empty or loaded with CUR) was achieved utilizing surfactant mixture of VE-TPGS and nonionic triblock copolymer, specifically poloxamer 407/Lutrol F-127 (LF-127), in 30:70 wt % ratio (Figure 2B). Owing to its temperature dependent self-assembly behavior (exhibited at low phase inversion temperature, PIT < 50 °C) and thermo-gelling behavior, LF-127 facilitated successful low-energy emulsification of our VE-rich oil phase, when combined with co-surfactant, VE-TPGS, analogous to earlier nano-self emulsifying drug delivery systems for VE in general, and T3 in particular [10,12]. In fact, the employed polyethylene glycol(PEG)-based surfactant mixture, composed of both VE-TPGS and LF-127, would further increase the stability of produced NE in external aqueous phase, owing to extended steric PEG chains on the NE droplet surface. Therefore, reduced viscosity of oily VE phase blend (30% IPM in Tph or δ-T3) combined with surfactant mix ratio of 70/30 (as LF-127:VE-TPGS), contributed towards efficient production of prototype VE-enriched NE platforms for CUR, via our lower energy hybrid homogenization process (Figure 1A,B) [10,12].

Maximizing the efficiency of CUR encapsulation in our bioactive prototype δ-T3 NE platform was analyzed, in comparison to inert Tph NE (Figure 2B). While both types of VE NE were quite comparable, in terms of either the incorporation efficiency (IE%) of CUR or the calculated drug loading capacity (LC%), it was evident that maximal IE% was maintained (approx. 97.5%–98.3%) when the added CUR amount was ≥2.0 mg, followed by incremental decrease with additional CUR amounts, to reach <40% at 5 mg of CUR. Conversely, the calculated LC% proportionally increased with admixed drug, until it reached a peak value ≈0.98%, also when CUR was added at 2.0 mg or higher to either of the NE VE-blended oil matrices.

Consequently, the adopted optimal VE-based NE formulations for CUR was based on 2.0 mg of CUR to start off as a feed drug into the oil matrix, which resulted in the highest initial entrapment efficiency (EE% at *D*_0_) of the drug, at 97.4% and 98.2% in Tph NE and δ-T3 NE prototypes, respectively (Table 1).

### 2.2. Determination of In Vitro Pharmaceutical Stability and Release Properties of Developed NE Formulations of CUR

The physico-chemical properties of our lead CUR-loaded δ-T3 NE were studied, via measuring not only the physical parameters of relative intensity of mean droplet size (in nm), average ζ-potential (in mV), plus their polydispersity index (PDI), but also the chemical stability of encapsulated drug, CUR, through EE% measurements after 60-days of cold storage (Table 1). Overall, our proof-of-concept formulations exhibited homogenous nano-scale droplets, with ave. negative ζ-potential ranging between −29.8 and −35.1 mV, and approx. PDI of 0.23–0.27, for both empty and CUR-loaded VE-based nanocarriers. On the other hand, the initial mean particle size of Tph NEs (empty or CUR-loaded) was significantly larger (about 1.4× bigger) than those of the corresponding δ-T3 NEs, in terms of CUR-loading. As observed in earlier, similar reports, it seems the flexibility and free rotation of saturated phytyl side chains potentially reduced the intermolecular forces between polar chromane rings of Tph, within the blended oil core of these NEs (Figure 1) [28]. Additionally, while both Tph-NE and δ-T3 NE platforms demonstrated superior CUR entrapment efficiency overall, EE% at *D*_0_ > 97%, the δ-T3 NE system maintained markedly higher EE% of CUR, in comparison with Tph-NE, as determined post-60 days of cold-shelf storage (*p* ≤ 0.05) (Table 1). This noted pharmaceutical stability of CUR-loaded δ-T3 NE would suggest the unsaturated phytyl residues of δ-T3 can mediate better arrangements and even tighter packing of the planar aromatic phenolic rings of CUR within the core chromane structures of δ-T3 oily matrix of NE (Figure 1) [10,12,28].

**Figure 3:**
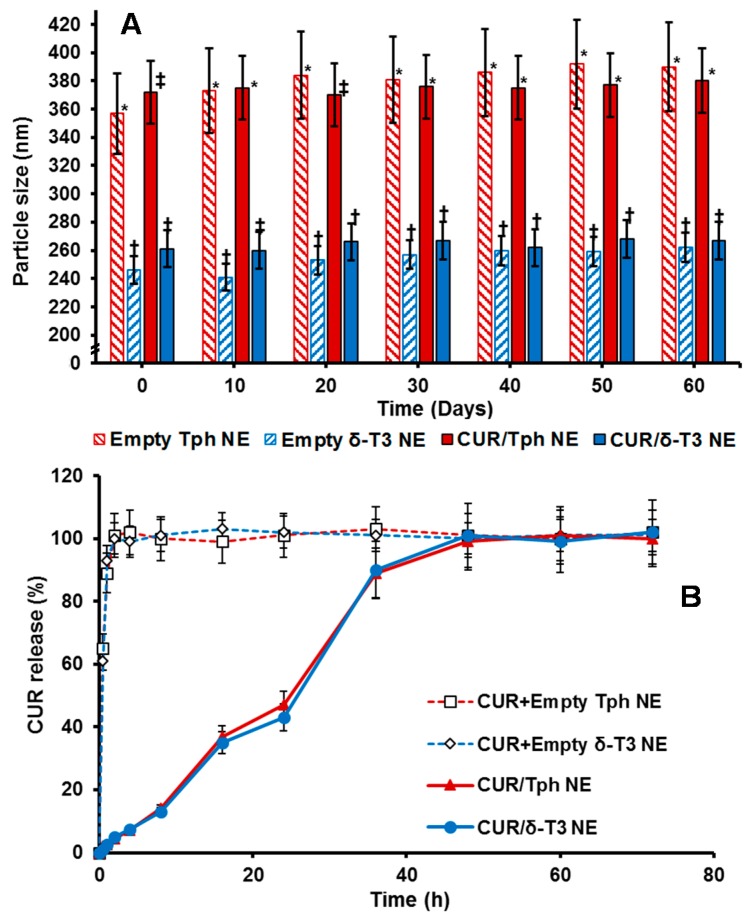
Shelf-stability and in vitro release of developed tocotrienol nanoemulsions of curcumin, showing (**A**) change in mean droplet size for drug-loaded and empty nanoemulsions over 60 days of cold storage; and (**B**) time-dependent release profile of encapsulated curcumin from nanoemulsions, in comparison to simple admixed preparations, under simulated sink conditions (*n* = 4–5, mean ± SD, mean values denoted with unlike symbols are statistically different, *p* ≤ 0.05).

Overall, the physical properties of our proof-of-concept NE platforms for CUR remained fairly unchanged over 8 weeks of refrigerated storage (Figure 3A), indicating efficiently-solubilized CUR-loaded δ-T3/Tph nano-droplets. Although the significant difference in droplet size between Tph-NE and δ-T3 NE platforms was consistent, the marginal size differences initially detected between empty and CUR-loaded δ-T3 NE were virtually equalized, after 30 days. The noted slight expansion in empty δ-T3 NE droplet diameter suggests a mutual stabilization interaction between CUR and δ-T3 molecules, within the oily phase of our prototype NE system for CUR [10,12].

A remarkably fast, almost immediate, in vitro release of CUR from the extemporaneous mixing of CUR with already fabricated empty Tph and δ-T3 NE vehicles, was displayed in contrast to the extended drug release profile supported by our prepared porotype Tph and δ-T3 NEs (Figure 3B). Such slow and controlled leakage of CUR (drug retained >50%, after 24 h) from both Tph and δ-T3 incorporating NEs confirms the firm and stable incorporation of CUR within the Tph and δ-T3 cores of our NE formulations.

### 2.3. Biocompatibility and Hemolysis Testing of Prototype δT3-NE Platform for CUR

**Figure 4:**
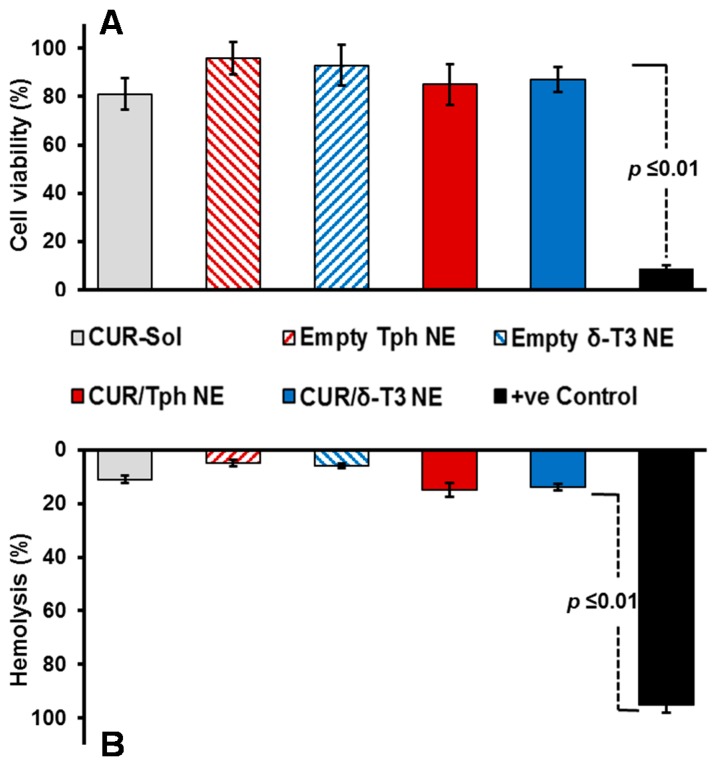
In vitro biocompatibility and hemocompatibility tests of prototype tocotrienol nanoemulsions of curcumin, evaluated (**A**) using murine subcutaneous connective tissue fibroblast (L929) cell culture model, post-48 h incubation with samples at CUR-equivalent concentration of 10 μM; and (**B**) for hemolysis degree determined after incubation of samples (equivalent to 5 μM approx. CUR physiological concentration) with red blood cells for 3 h, at 37  °C (*n* = 4, mean ± SE).

Our proof-of-concept δ-T3 NE is proposed as an active nano-pharmaceutical formulation loaded with CUR, intended for systemic administration, and therefore was evaluated in vitro, using L929 cell culture-based biocompatibility safety assessment (Figure 4A), which is derived from ISO-10993/FDA draft guidance for submicron and nanotechnology components. At physiologically relevant CUR concentration up to 10 μM, both drug-loaded Tph and δ-T3 NEs—along with empty vehicle and free CUR-sol controls—effectively passed the standardized biocompatibility screen, with over 80% cell viability, following 48 h co-incubation with normal connective tissue fibroblasts, conceivably owing to their superior pharmaceutical properties and stability [19,25,29].

Further verification of the pharmaceutical suitability of our lead CUR-loaded δ-T3 NE for systemic application was attained via biocompatibility assay in the presence of red blood cells (RBCs) by measuring hemolysis (Figure 4B). Overall, data indicated that all treatments produced minimal (15.6%–19.3%) hemolysis of RBCs overall, in comparison to positive control applied under the same test conditions. Albeit, as commonly implicated with applied chemo-active compounds, CUR-containing NE were marginally on the higher end of hemolysis values (17.5%–19.3% ≈ free CUR-Sol), relative to their respective drug-free NE vehicles. Effectually, our developed δ-T3 NE platform, either alone or loaded with CUR, demonstrated good bio- and hemo-compatibilities as appropriate for therapeutic systemic administrations [29].

### 2.4. Evaluation of NF-κB Pathway Inhibition and Apoptosis Induction by Combination CUR in δT3-NE

The main pharmacologic advantage of designing a prototype δ-T3 NE platform of CUR lies in its ability to efficiently co-deliver both anti-cancer compounds into malignant cells, in order to jointly inhibit the NF-κB pro-survival signaling pathway, which is implicated in cancer cell proliferation, survival, angiogenesis and metastasis [15,16]. Additionally, together δ-T3 + CUR can prominently induce pro-apoptotic machinery, which would ultimately boost the overall anti-neoplastic efficacy of such combined NE treatment [13,30].

**Figure 5:**
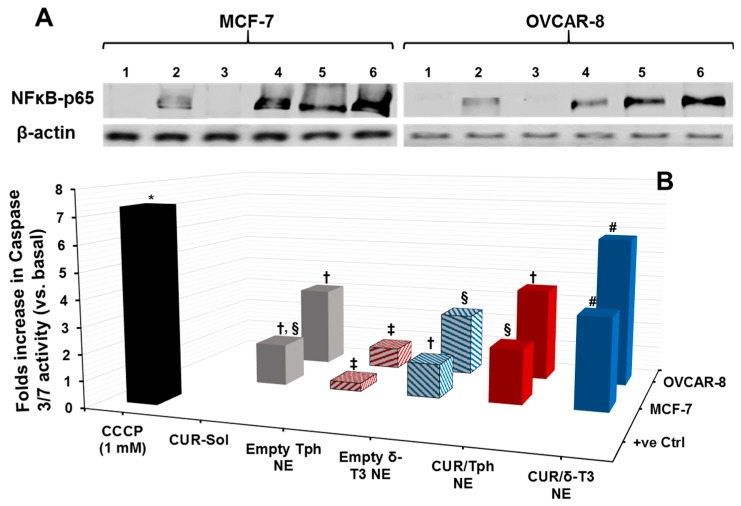
Evaluation of NF-κB pathway inhibition and apoptosis induction. Both test cancer cell models, MCF-7 and OVCAR-8, were treated with various preparations at 25 μM of CUR-equivalent concentration, and assayed utilizing (**A**) western blot analysis of inhibition of cytosolic NF-κB, following treatment with different samples. For each treated cancer cell line, lanes 1, 2, 3, 4, 5, and 6 represent untreated control, CUR-Sol, empty Tph NE, empty δ-T3 NE, CUR/Tph NE, CUR/δ-T3 NE, respectively. A total of 30 and 20 μg of protein extracts were loaded per well for MCF-7 and OVCAR-8 cells, respectively, and β-actin served as a loading control (*n* = 3); and (**B**) microplate-based fluorimetric caspase 3/7 activation measurement kit, following 24 h of co-incubation at 37 °C, 5% CO_2_ conditions, and (*n* = 3–4, mean ± SE values denoted with unlike symbols are statistically different, *p* ≤ 0.05).

Cytosolic protein extracted from human breast (MCF-7) and ovarian (OVCAR-8) carcinoma cells, treated with 25 μM equivalent concentration of CUR in different formulations, was evaluated by Western blots—using anti-NF-κB p65 antibody—to qualitatively estimate the inhibition level of the NF-κB pathway (Figure 5A). Normally, the canonical pathway of NF-κB activation involves phosphorylation of serine residues in the signal responsive region (SRR) of classical inhibitor of NF-κB proteins (IκBs), leading to IκB ubiquitination and degradation. This results in release of the NF-κB dimers (mostly containing the p56 subunit to be detected by anti-NF-κB p65 antibody), which can then translocate to the nucleus and induce transcription of target genes [30]. Hence, indistinct NF-κB bands at 64 kDa were observed in untreated cells and also in cells treated with empty Tph-NE, which has no inhibitory effect on NF-κB nuclear translocation. Conversely, the darker bands detected in CUR-Sol, CUR/Tph NE, and δ-T3 NE treated cells (either MCF-7 or OVCAR-8) affirmed the presence of more inactive NF-κB heterodimers (visualized through NF-κB p56 subunit) in cytosol, which can be directly attributed to the activity of each agent of applied agents, CUR, and δ-T3. It became obvious that CUR/δ-T3 NE further intensified the NF-κB p56 bands, in both test cell models, compared to other treatments, indicating that our prototype NE system was quite efficient in co-delivering both active compounds CUR and δ-T3 into tumor cells [15].

As one of the most important mechanisms of tumor cell death, triggered intracellular apoptosis markers were investigated following 24 h of exposure to different CUR and δ-T3-containing treatments. Figure 5B reveals increased apoptotic induction and marked activation of effector apoptosis caspase proteins (caspase-3 and -7), associated with cancer cell exposure to δ-T3 NE and CUR-containing formulations, but not with empty Tph NE. Most importantly, the significant increase in caspase-3/-7 levels was about 1.8–2.3 folds higher in breast (MCF-7) cancer cells treated with the combined CUR/δ-T3 NE, compared to those in cells treated with empty δ-T3 NE and CUR in Tph NE, respectively.

Following a similar fashion in ovarian (OVCAR-8) cancer cells, the prominent upsurge in caspase-3/-7 levels with our prototype CUR/δ-T3 NE was nearly comparable to that induced by CCCP positive control, and was almost 1.65–2.2 folds higher than empty δ-T3 NE and CUR in Tph NE treatments, respectively. Such superior activation of influential caspase proteins (3 and 7)—commonly recognized as chief effector caspases implicated in driving apoptosis pathways in tumors—firmly elucidate an amplified apoptosis induction role of CUR in δ-T3 NE as a major underlying anti-cancer mechanism [21,30].

### 2.5. Anticancer Efficacy of CUR-Combined δT3-NE System against Cultured Human Carcinomas

**Figure 6:**
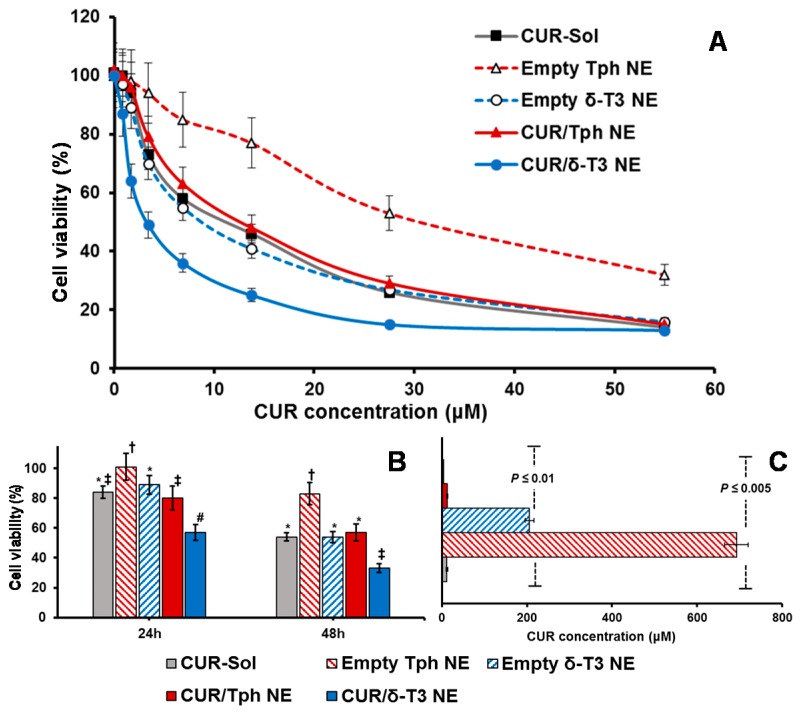
Concentration- and time- dependent cytotoxicity against MCF-7 cell cultures. Microplate-based CellTiter Blue^®^ assay of human breast carcinoma cells, MCF-7 cells, measured for different empty and CUR-loaded VE- nanoemulsions vs. CUR solution serving as free drug control (**A**) at CUR-equivalent concentration range of 0.85–55 μM after incubation for 48 h; or (**B**) at “cross-section CUR-equivalent concentration of 25 μM, over co-incubation period of 24–48 h; along with (**C**) IC_50_ values calculated for each preparation type, after 48 h treatment, at 37 °C, 5% CO_2_ conditions (*n* = 3–4, mean ± SE, mean values denoted with unlike symbols are statistically different, *p* ≤ 0.05).

**Figure 7:**
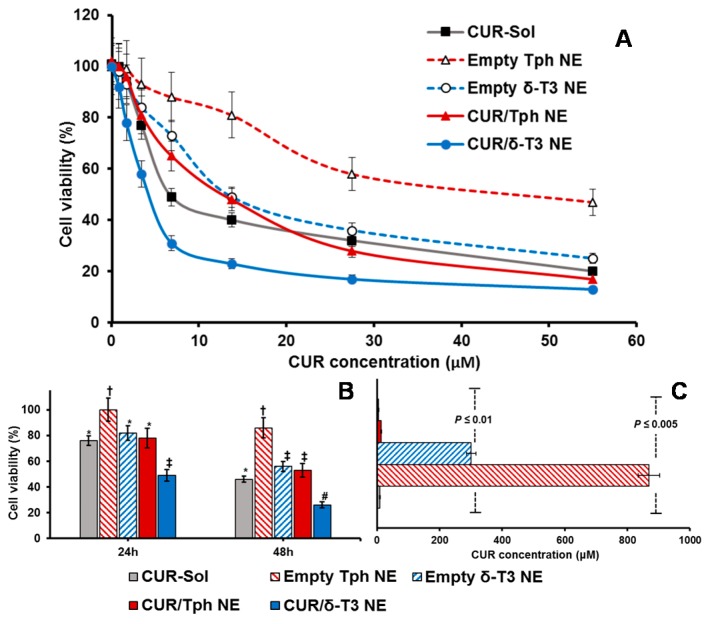
Concentration- and time-dependent cytotoxicity assays against OVCAR-8 cell cultures. Microplate-based CellTiter Blue^®^ assay of human ovarian adenocarcinoma cells, OVCAR-8 cells, measured for different empty and CUR-loaded VE-nanoemulsions vs. CUR solution serving as free drug control (**A**) at CUR-equivalent concentration range of 0.85–55 μM after incubation for 48 h; or (**B**) at “cross-section” CUR-equivalent concentration of 25 μM, over co-incubation period of for 24–48 h; along with (**C**) IC_50_ values calculated for each preparation type, after 48 h treatment, at 37 °C, 5% CO_2_ conditions (*n* = 3–4, mean ± SE, mean values denoted with unlike symbols are statistically different, *p* ≤ 0.05).

An expanding body of evidence corroborates the antiproliferative activity of CUR and δ-T3 against various tumor, but not normal, cells. Therefore, our combined CUR/δ-T3 NE was likely to mediate a strong and direct tumor cell arrest. Utilizing cultured human breast (MCF-7) and ovarian (OVCAR-8) cancer cells (Figure 6 and Figure 7, respectively), the cytotoxicity of empty and CUR-loaded δ-T3 NE was examined, in both concentration and time-dependent protocols. Figure 6A demonstrated the pronounced cytotoxic profile of CUR/δ-T3 NE against MCF-7 cells, over almost the entire tested drug concentration range, vs. all other controls, achieving a maximal cell kill (>90%) at <30 µM of CUR. The calculated IC_50_ value for each applied treatment (Figure 6C) revealed that CUR/δ-T3 NE was three to four times more potent than any CUR-containing formulation (either free or loaded in Tph NE), and 49 times more effective than empty δ-T3 NE preparation (based on δ-T3 within NE). Furthermore, a time-dependent profile of anti-proliferative activities (Figure 6B) showed that our prototype CUR/ δ-T3 NE exhibited the lowest tumor cell viabilities (cal. 57.2% and 33.4% after 24 and 48 h of exposure, respectively), when compared to all treatment controls. While Tph NE vehicle control appeared to exhibit marginal anti-cancer activity, when measured at both co-incubation times, and tested CUR-equivalent concentrations, the treatments CUR-Sol, empty δ-T3 NE and CUR/Tph NE appeared to have comparable activities against MCF-7 cells.

Analogously, data shown in Figure 7A–C established that prototype CUR/δ-T3 NE produced the strongest antineoplastic profile against OVCAR-8 cells with the lowest IC_50_ value (compared to all other control and drug-containing treatments), determined after 48 h of co-incubation. The growth of OVCAR-8 cancer cells was most significantly arrested by CUR/δ-T3 NE treatment, at 49.1% and 26.4%, when measured post-24 h and -48 h of co-incubation, respectively.

Finally, based on combination index (CI) ratios calculated for CUR loaded in δ-T3 or Tph NE preparations, the prototype CUR/δ-T3 NE combo was found to yield effective synergism of in vitro anticancer efficacy (CI = 0.814, vs. 1.862 for CUR/Tph NE), against human ovarian carcinoma cultures. Also, in breast cancer cells, the combination CUR/δ-T3 NE produced even stronger synergism of antineoplastic activity (CI = 0.692, vs. 1.493 for CUR/Tph NE), altogether corroborating the augmented anti-cancer effectiveness of our CUR/δ-T3 NE system, evident in both tested human cancer models [31].

## 3. Materials and Methods

### 3.1. Materials

Commercial GMPO-grade Annatto δ-tocotrienol (δ-T3) “Delta Gold^®^” was generously provided by the manufacturer, American River Nutrition (Hadley, MA). Delta Gold^®^ is non-GMO certified Tph-free Vitamin E product which contains 100 wt % of T3s (≥90% as δ-T3 and ≤10% as γ-T3). Glycerol, isopropyl myristate (IPM), carbonyl cyanide m-chlorophenylhydrazone protonphore reagent (CCCP), Trypsin/EDTA, penicillin/streptomycin, and fetal bovine serum (FBS) were obtained from Fisher Scientific (Waltham, MA, USA). Amicon Ultra-15 centrifugal filters and flat/clear-bottom BD-Black 96-well tissue culture plates were purchased from VWR International (Radnor, PA, USA). Antares Health Products Inc. (Batavia, IL, USA) generously donated the NF-grade Vitamin E-TPGS (d-α-tocopheryl polyethyleneglycol succinate, VE-TPGS), while NF-grade Lutrol^®^ F127/Poloxamer 407 (polyoxyethylene-polyoxypropylene block copolymer, *M*_wt_ = 12,600 g/mol, LF-127) was generously supplied by Muchler Inc./BASF (Cincinnati, OH, USA). Murine subcutaneous connective tissue fibroblasts (L929) cell line, along with human breast carcinoma (MCF-7) were purchased from American Type Culture Collections (Manassas, VA, USA), while the human ovarian adenocarcinoma cells (OVCAR-8) were obtained from National Cancer Institute (NCI/DCTD) Repository (Bethesda, MD, USA). Ultrapure Milli-Q (MQ) water was utilized for all preparations, unless indicated otherwise.

### 3.2. Methods

#### 3.2.1. Formulation of Tocotrienol-Rich Nanoemulsions and Curcumin Incorporation

δT3-based NEs were prepared by novel hybrid homogenization technique (under N_2_ gas) [12]. Briefly, to the T3-rich oily phase (composed of IPM-blended δ-T3), the surfactant mixture (Smix) of LF-127:TPGS in 70:30 wt % ratio was added, while warming and gently mixing on a vortex mixer at 800 rpm at 50 °C, for 2 min. For CUR-loaded formulation, appropriate volume of CUR stock solution in organic solvent (15 mmol of CUR dissolved per mL of 9:1 *v*/*v* of chloroform: ethanol) was vortex-mixed with the homogenous oil-surfactant mixture, produced in the previous step, then, cosolvent was evaporated under vacuum (100 mTorr) using rotary-evaporator (at 35 °C, Labconco, Kansas City, MO, USA) [25]. After verification of translucent and homogenous mixture clear of any precipitates, it was hydrated with MQ water—containing 2.25 wt % glycerol, with adjusted pH~7.6 ± 0.03, for tonicity—followed by brief vortex mixing, then homogenization for 5 min at 20,000 rpm using Ultra Turrax-10 homogenizer (IKA Works Inc., Wilmington, NC, USA) to produce the coarse oil-in-water (O/W) emulsion, which was then passed, once at 300 psi, through 0.4 μm Nuclepore^®^ polycarbonate filters (GE Healthcare Bio-Sciences, Pittsburgh, PA, USA) via a 1XT Lipex mini-thermo-barrel extruder (Transferra Nanosciences Inc., Burnaby, BC, Canada). Subsequently, micro-emulsions were brought, rapidly and with agitation, from the corresponding hydrophilic-hydrophobic balance (HLB) temperature (approx. 50 ± 3 °C) to 25 °C, using water/ice bath. Following this single phase inversion temperature (PIT) cycle, the produced NEs were ultra-sonicated at 4-watts power, using a Misonix XL2000 probe sonicator (P-6 low amplitude microprobe, Qsonica, Newtown, CT, USA) for only 15 min, to obtain homogenous nanosized oil droplets [10,12,26].

#### 3.2.2. Physicochemical Characterization of NEs

##### Droplet Size Analysis

Prototype drug free and CUR-loaded NEs were diluted with MQ-water before analysis, and the average oil droplet hydrodynamic diameter (*z*-Ave) and the polydispersity index (PDI) were determined using dynamic light scattering technique, via Malvern Zetasizer Nano-ZS (Malvern Instruments Inc., Westborough, MA, USA) [12,25].

##### Zeta Potential (ζ) Measurements

All NEs samples were diluted with MQ-water (typical pH~5.8) and placed in the electrophoretic cell of the Malvern Zetasizer Nano-ZS (Nano ZS, Malvern Instruments Inc., Westborough, MA, USA) and the average surface charge was determined [12,25].

#### 3.2.3. Incorporation Efficiency (IE%) and Loading Capacity (LC%) of CUR in Lead Formulations

High-performance liquid chromatography (HPLC) analytical method was used to determine the levels of CUR incorporated in the selected NE. Formulation samples (20 µL) were injected through ACE PFP C-18 column (4.6 × 250 mm, 4 µm packing vol.) using degassed acetonitril:0.1% trifluoroacetic acid in H_2_O (45:55 *v*/*v*) as mobile phase. The flow rate of the mobile phase was maintained at 1 mL/min, at 25 °C, and UV-detection was performed at 428 nm. The CUR concentration in the sample was determined using a calibration curve of CUR dissolved in methanol. The HPLC method showed good reproducibility, with precision of less than 5% RSD, as well as excellent accuracy between 90.7% and 96.85% for CUR. The lower limit of CUR quantitation was 0.05 µg/mL. The initial incorporation efficiency of CUR (IE%, calculated using Equation (1), below) was determined by ultra-filtration technique, using centrifugal filter devices (*M*_w_CO = 5000 Da; Amicon Ultra-15, Millipore, Bedford, MA, USA), followed by HPLC quantification [6,30,32]:

IE% = [(*D*_i_ − *D*_f_)/*D*_i_] × 100 (1)

where “*D*_i_” is the amount of initial CUR added and “*D*_f_” is the amount of CUR detected in sample after centrifugation.

Similarly, The Loading Capacity of CUR (LC%, calculated using Equation (2), below) was determined, right after final NE preparation (*t*_0_) [6,21,29]:

LC% = [(*D*_i_ − *D*_f_)/*O*] × 100 (2)

where “*O*” is the amount of initial oily phase added to form each NE.

#### 3.2.4. Storage Stability

Similar to earlier shelf-storage stability and stress testing performed to pharmaceutically characterize T3-based NE fabricated using the hybrid nanoemulsification process [12], all empty and CUR-loaded prototype δ-T3 and Tph NEs were monitored, over 2 months of storage, under refrigerated conditions (4–8 °C), for time-dependent changes in the physical characteristics (drug precipitation, change in micelle size, and surface charge) of the formulations. The chemical stability of CUR in formulations after 60 days (*t*_60_), using optimal encapsulation of CUR amount, initially added at 2 mg into NE formulations (*t*_60_), was evaluated by the HPLC as described above, expressed as entrapment efficiency, EE% for each time point, as follows [12,28,29].

EE% = [(*D*_NE_)/*D*_Feed_] × 100 (3)

where “*D*_NE_” is the weight of CUR determined in each formulation, and “*D*_Feed_” is the weight of added CUR feed, optimized at 2 mg.

#### 3.2.5. In Vitro Release Studies at “Sink” Conditions

To study the time-dependent release of CUR—from either δ-T3 or Tph NEs compared to non-emulsified drug—the dialysis bag method was utilized. Drug-loaded samples (0.2 mL of approx. 3 mM of Brb) were placed in Spectra/Por^®^ dialysis cellulose ester (CE) membranes (*M*_w_CO = 25 kDa, Spectrum Laboratories Inc., Rancho Dominguez, CA, USA), after soaking overnight in release medium. Closed dialysis bags were submerged into 200 mL of test external release medium of phosphate-buffered saline solution (PBS, pH 7.4), containing 0.05% of Tween 80, in order to maintain pseudo-sink conditions in a limited volume, up to 72 h of incubation with continuous stirring at 200 rpm speed. At specific time intervals, 0.5 mL samples of each release medium were withdrawn, and replaced with an equal volume of fresh medium, then samples were filtered through 0.22 µm syringe membrane filters. The CUR content in samples was finally determined by HPLC, as described above [12,25].

#### 3.2.6. In Vitro Cell Culture

Human breast carcinoma (MCF-7) and human ovarian adenocarcinoma cells were grown in complete Dulbecco-modified MEM and RPMI-1640 culture media, respectively, while subcutaneous connective tissue mouse fibroblasts (L929) were grown in complete Eagle’s MEM culture medium—prepared by adding 10% fetal bovine serum and penicillin (100 U/mL)/streptomycin (100 μg/mL)—in a humidified environment of 37 °C, 5% CO_2_. Cells were seeded at a concentration of 6 × 10^3^–1.5 × 10^4^ cells/cm^2^ and sub-cultured at approximately 70%–80% confluency. Cells between passages 5–10 were used for experimentation [19].

#### 3.2.7. In Vitro Biocompatibility and Hemocompatibility Assays

##### Biocompatibility Screen

Murine subcutaneous connective tissue fibroblasts (L929) were seeded at 10 × 10^3^ cells per well of 96-well plates in triplicates for 24 h. Then, the respective complete culture media were exchanged for serum-free media only (serving as the no-treatment cell culture negative control, −ve ctrl), or containing several two-fold serial dilutions of free CUR dissolved in hydro-alcoholic solution (CUR-Sol), compared with empty δ-T3 and Tph NE vehicles and corresponding CUR-loaded prototype NE formulations. Finally, after 48 h of co-incubation, total L929 cell viability was determined using CellTiter Blue^®^ Kit (Promega, Madison, WI, USA), after washing twice with Hank’s Balanced Saline solution, pH 7.4 (HBSS) and reading sample plate fluorescence (λ_Ex_ = 480 nm/λ_Em_ = 530 nm), using Synergy 2 Biotek fluorescence plate reader (Biotek instruments Co., Winooski, VT, USA), according to the manufacturer’s instructions [19,32].

##### In Vitro Hemolysis Assay

Hemolysis studies were carried out for free CUR-Sol, empty δ-T3 and Tph, NEs along with test CUR-containing samples, according to previous reports [33]. The release of hemoglobin from the erythrocytes was used for toxicity measurements of these carriers. Briefly, Defibrinated rabbit blood (Thermo Scientific, Waltham, MA, USA) was diluted 10 times with PBS and centrifuged at 2000 rpm for 15 min. The supernatant was decanted, and the precipitate was rinsed three times with PBS, followed by centrifugation at 2000 rpm for 15 min. The concentration of resulting blood cells was adjusted to 2% (*v*/*v*). At a physiologically relevant dilution scenario of IV bolus administration (5 µg/mL of CUR-equivalent concentration), 50 µL of test samples was mixed with 500 μL of blood cells, and the resulting suspensions were incubated at 37  °C for 3 h. The samples were then centrifuged at 2000 rpm for 15 min. The absorbance of the supernatant was measured at 540 nm to determine the amount of hemoglobin release. Zero hemolysis and 100% hemolysis consisted of red blood cells suspended in physiological saline (−ve ctrl) and distilled water (+ve ctrl), respectively.

The percentage of hemolysis was determined as per the following equation:

(4) $$\mathrm{Hemolysis}\;\left(\%\right)=\;\frac{\left(A_{\mathrm{ts}}-A_{0}\right)}{A_{100}-A_{0}}\times 100$$

where, *A*_ts_ is the absorbance of the test sample, *A*_100_ is the absorbance of completely lysed red blood cells in distilled water, and *A*_0_ is the absorbance of zero hemolysis [29,33].

#### 3.2.8. Apoptosis Assays

Approximately 1.5 × 10^6^ cells—used from each of MCF-7 or OVCAR-8 human breast and ovarian carcinoma, respectively—were grown overnight to attach to 25 cm^2^ flasks. Cells were incubated for 24 h with CUR-loaded NE and blank δ-T3 and Tph NEs vehicle control, along with free drug solution control (CUR-Sol), at a concentration equivalent to 25 µM of drug. In addition, CCCP was added to induce apoptosis as positive control for the same period and incubation conditions. Cells were then washed, trypsinized and collected, then normalized by their protein content, determined as per standard BCA protein assay kit protocol (Thermo Fischer Scientific, Rockford, IL, USA). Afterwards, 100 μL of the Apo-ONE™ Homogenous caspase 3/7 assay substrate solution was added to each well containing 100 μL of sample media, and after 30 s of mixing, the plate contents were incubated for 3 h. Finally, the sample plate fluorescence values were measured (λ excitation/emission: 480 nm/530 nm) using Synergy 2 Biotek fluorescence plate reader (Biotek instruments Co., Winooski, VT, USA), according to manufacturer’s instructions. The caspase-3/7 activity was reported as percentage (%) activation relative to untreated control [10,32].

#### 3.2.9. Evaluation of NF-κB Activity by Western Blotting

Human MCF-7 and OVCAR-8 cancer cells growing in T-25 flasks were treated with 25 μM concentration of free CUR-Sol solution, along with CUR-loaded NEs formulations and blank δ-T3 and Tph NEs vehicle control for 24 h. Then, cells were lysed and the total protein was extracted and quantified by BCA protein assay kit. Subsequently, protein was separated on a 4%–15% SDS-PAGE gradient gel and then transferred onto nitrocellulose membrane. Nitrocellulose membrane blots were incubated with anti-NF-κB-p65 antibody, or anti-β-actin monoclonal antibody overnight at 4 °C, followed by 1 h incubation at R.T. with a horseradish peroxidase-conjugated secondary antibody. To visualize the protein of interest (i.e., cytosolic NF-κB, 64 kDa) along with internal control (β-actin, 42 kDa), the membranes were then incubated for 5 min with enhanced chemiluminescent substrate and the exposed film was finally imaged using a Kodak Gel-logic 200 imaging system (Carestream Health, Rochester, NY, USA) [15,30].

#### 3.2.10. Cytotoxicity Assay

Human breast adenocarcinoma (MCF-7) along with human ovarian cancer (OVCAR-8) cells were seeded at 10 × 10^3^ cells per well, in 96-well flat-bottom black microplates in four replicates for 24 h. Then, respective complete culture media were exchanged for serum-free media (SFM) containing several two-fold serial dilutions of un-encapsulated CUR-Sol as positive drug control treatment, and drug-equivalent concentrations of CUR-loaded NE formulation, plus blank vehicle controls of δ-T3 and Tph NEs, all compared with plain SFM serving as negative control (−ve ctrl). Following 48 h of co-incubation at 37 °C, in 5% CO_2_, the total cell viability was determined using CellTiter Blue^®^ cell viability assay Kit (Promega, Madison, WI, USA) after washing twice with HBSS and reading sample plate fluorescence (λ excitation/emission: 550 nm/590 nm) and using Synergy 2 Biotek fluorescence plate reader (Biotek instruments Co., Winooski, VT, USA) according to the manufacturer’s instructions [10,32].

In addition, the combination index (CI) of CUR and δ-T3 NE or Tph NE was determined using the classic isobologram equation of Chou and Talalay [31]:

CI = *a*/*A* + *b*/*B* (5)

where *a* is IC_50_ of CUR in combination with each NE at concentration *b*, i is the IC_50_ of CUR alone, and *B* is the IC_50_ of each NE in the absence of CUR. Based on this method, when CI < 1, the interaction is synergistic; when CI = 1, the interaction is additive; and when CI > 1, the two agents are antagonistic [30,31].

#### 3.2.11. Data Analysis

A minimum of triplicates were run for each experiment, unless indicated differently. Data were reported as mean ± standard error (SE), unless noted otherwise. Comparisons between two groups were made using Student’s *t*-test, and for more than two groups, Kurskal-Wallace test with Tukey’s post-hoc analysis, was used to compare results. The *p* < 0.05 values are considered statistically significant. All statistical analyses were performed using GraphPad Prism^®^ software, ver. 5.0 [12,26,32].

## 4. Conclusions

Developed tocotrienol-based nanoemulsion delivery systems, recognized to arrest cancer growth and induce cellular apoptosis, were utilized to enhance the in vitro therapeutic activity of curcumin against human breast and ovarian tumor cells. Above and beyond the superior nanoscale properties and pharmaceutical stability obtained for curcumin loaded into our prototype δ-tocotrienol nanoemulsion, this combination strikingly suppressed constitutive NF-κB activation and induced effector caspases, mediating extensive cancer apoptosis. Our proof-of-concept combination of curcumin in δ-tocotrienol nanoemulsion has effectively generated remarkable synergism in anti-neoplastic efficacy overall, in concentration and time-dependent manners, as evaluated in both test human cancer cell types. Taken together, our current positive data can substantiate further development and pre-clinical investigation of such therapeutic combinations of curcumin-loaded tocotrienol nanoemulsion systems for potential applications in clinical cancer therapy.

## Figures and Tables

**Table 1:** Physico-chemical properties and stability of developed CUR-NE preparations (*n* = 3–4, mean values denoted with unlike symbols are statistically different, *p* ≤ 0.05).

NE Formula	CUR	Mean Particle Size (nm ± SD)	PDI	ζ-Potential (mV ± SD)	EE% (±SD) in *D*_0_	EE% (±SD) in *D*_60_
α-Tph NE	Empty	358.4 ± 28.6 *	0.228 *	−29.68 ± 1.5 *	N/A	N/A
	+	367.8 ± 32.3 ^†^	0.266 ^†^	−31.20 ± 1.9 *	97.4 ± 5.8	92.8 ± 6.9 *
δ-T3 NE	Empty	239.6 ± 38.4 *	0.251 ^‡^	−33.27 ± 2.3 ^†^	N/A	N/A
	+	261.5 ± 30.5 ^‡^	0.273 ^†^	−35.15 ± 2.5 ^†^	98.2 ± 6.5	97.7 ± 5.8 ^†^
